# Workplace Racial Composition Explains High Perceived Discrimination of High Socioeconomic Status African American Men

**DOI:** 10.3390/brainsci8080139

**Published:** 2018-07-27

**Authors:** Shervin Assari, Maryam Moghani Lankarani

**Affiliations:** 1 Department of Psychiatry, University of Michigan, 4250 Plymouth Rd., Ann Arbor, MI 48109-2700, USA; lankaranii@yahoo.com; 2 Department of Psychology, University of California, Los Angeles (UCLA), Los Angeles, CA 90095, USA; 3 BRITE Center for Science, Research and Policy, University of California, Los Angeles (UCLA), Los Angeles, CA 90095, USA; 4 Center for Research on Ethnicity, Culture and Health (CRECH), School of Public Health, University of Michigan, Ann Arbor, MI 48104, USA

**Keywords:** African Americans, blacks, ethnic groups, racism, discrimination, socioeconomic status (SES), income

## Abstract

Background: Sociological and epidemiological literature have both shown that socioeconomic status (SES) protects populations and individuals against health problems. Recent research, however, has shown that African Americans gain less from their SES and African Americans of high SES, particularly males, may be vulnerable to perceived discrimination, as explained by the Minorities’ Diminished Returns theory. One potential mechanism for this phenomenon is that high SES African Americans have a higher tendency to work in predominantly White workplaces, which increases their perceived discrimination. It is, however, unknown if the links between SES, working in predominantly White work groups and perceived discrimination differ for male and female African Americans. Aim: To test the associations between SES, workplace racial composition and perceived discrimination in a nationally representative sample of male and female African American adults. Methods. This study included a total number of 1775 employed African American adults who were either male (*n* = 676) or female (*n* = 1099), all enrolled from the National Survey of American Life (NSAL). The study measured gender, age, SES (educational attainment and household income), workplace racial composition and perceived discrimination. Structural Equation Modeling (SEM) was applied in the overall sample and also by gender. Results: In the pooled sample that included both genders, high education and household income were associated with working in a predominantly White work group, which was in turn associated with more perceived discrimination. We did not find gender differences in the associations between SES, workplace racial composition and perceived discrimination. Conclusion: Although racial composition of workplace may be a mechanism by which high SES increases discriminatory experiences for African Americans, males and females may not differ in this regard. Policies are needed to reduce discrimination in racially diverse workplaces. This is particularly the case for African Americans who work in predominantly White work environments.

## 1. Introduction

Although socioeconomic status (SES) has well-established protective effects on population health [1,2,3,4,5,6,7,8,9,10], less is known about the mechanisms by which high SES becomes a vulnerability factor. For instance, although we know that SES generates less or no mental health gain for racial and ethnic minorities [11,12,13,14] and particularly for African American men [15,16,17], the underlying mechanism for this phenomenon is unknown. High SES may even operate as a risk factor for poor mental health in African Americans [12,15,16,17,18,19,20].

Although the exact underlying mechanism for such a counter-intuitive phenomenon is still unknown, the increased exposure [12,21,22,23,24] or vulnerability [25] of high SES African Americans—particularly African American men—to perceived discrimination may be a potential explanation [26]. Perceived discrimination has a unique role given it causes poor health disparities due to low SES and race /ethnic minority status [27,28]. Given a wide range of non-specific effects, perceived discrimination increases a wide range of undesired physical and mental health outcomes [29,30,31] such as obesity [32,33], heart disease [34], hypertension [35,36], pain [37], disease management [38] and all-cause mortality [39]. Discrimination is also a risk factor for psychological problems and psychiatric disorders [40,41] such as symptoms of anxiety and depression [42], psychological distress [43], clinical depression [25], eating disorders [44], behavioral problems [45], substance use [46,47] and suicide [48,49]. If perceived discrimination is a reason for why SES resources do not generate the same health gain for African Americans as Whites, we should be able to document an increase in perceived discrimination in the lives of high SES African Americans. 

Although overall, SES resources (e.g., educational attainment and household income) are protective against poor health [50,51,52], the health gains that follow high SES are smaller or absent for minority populations [53] particularly African Americans [21,54] compared to Whites. Weaker effects of educational attainment are shown in drinking behaviors [55], smoking [39], diet [56], sleep [57], suicide [18] body mass index (BMI) [58] and life expectancy [55] for African Americans than Whites. One explanation is that due to labor market discrimination [59], educational attainment generates far more income and wealth for Whites than for African Americans [60,61]. This, however, cannot be the full explanation because income itself impacts unequal health gains for Whites and African Americans. The effects of income on mental health [13], chronic disease [62], obesity [63] and oral health [64] are all smaller for African Americans than Whites. Such patterns are shown for children [63], youth [17] and adults [11,13]. This robust pattern, which is also found for other SES indicators such as employment [65] and marital status [14,58] and holds for trans-generational effects [14,58]. An unexpected finding is that high SES may even increase the risk of mental health problems for African Americans. A higher risk of depression [12,26] and depressive symptoms [15,24] in African American males of high SES need more research. 

To explain the diminished health return of SES resources for African Americans [15,16,18], theory and research have competing theories that attribute diminished returns to economic, sociological and social psychological mechanisms. Supporting an economic explanation, empirical research has shown that education generates less income for African Americans than Whites [60,61]. This is also understandable given preferences and practices of labor market and availability of job market and lower quality of education in urban areas [66]. A sociological explanation emphasizes on the role of structural racism that minimizes the gains of SES resources for African Americans and other minority groups [54]. Race, as a social rather than a biological construct, is a proxy of access to the opportunity structure and treatment by society [10,67,68,69]. In statistical terms, structural racism shows its effect as differential slopes of resources across racial groups, with minorities and marginalized groups always being at a relative disadvantage compared to Whites for the effects of their resources on health outcomes [21,54]. Finally, a social psychological explanation proposes that increased inter-group contact results in higher discriminatory experiences for high SES African Americans, that in turn reduce the health gain from their SES resources [21]. That is, high SES African Americans live and work in predominantly White areas that may increase discrimination for African Americans [23,24]. 

Some empirical evidence exists regarding the psychological explanation. In the National Survey of American Life (NSAL)—Adults’ data, educational attainment was positively associated with high suicidal ideation among Caribbean Black females but not males [18]. In the American Changing Lives (ACL) data, high education credentials were positively associated with an increase in depressive symptoms over time for African American men but not women [15]. In another study among adults who participated in the NSAL, high household income was associated with higher risk of major depressive disorder (MDD) in African American men [12]. The increase in perceived discrimination of high SES African American men and women may explain the increases in the social, psychological and biological costs of upward social mobility for African Americans [15,18,55,57,65].

## 2. Aims

To better understand the role of workplace composition as a potential mechanism for high SES as a vulnerability factor for African American men, this study used a national sample of African Americans to explore the associations between SES, racial composition at workplace and perceived discrimination in African American men and women. This study hypothesized that there is a heterogeneity of the effects of SES indicators (household income and educational attainment) on racial composition at work and the effects of racial composition at work on perceived discrimination by gender, with males being at a disadvantage.

## 3. Methods

### 3.1. Design

This study used a cross-sectional design. We used data from the NSAL, 2003 [70,71,72]. As a part of the Collaborative Psychiatric Epidemiology Surveys (CPES), NSAL was funded by the National Institute of Mental Health (NIMH). CPES is the most comprehensive mental health survey focused on psychiatric epidemiology of diverse racial and ethnic groups in the United States.

### 3.2. Ethics

The University of Michigan (UM) Institute Review Board (IRB) approved the NSAL study protocol. Written informed consent was received from all the participants. Respondents were financially compensated for their time.

### 3.3. Participants and Sampling

The NSAL is a household survey of Blacks with a non-Hispanic White control group that lived in their proximity. The NSAL used a national household probability sampling to recruit participants. African American and non-Hispanic White sample in the NSAL were selected from large cities, other urban areas, or rural areas [70,71,72]. The NSAL used a multi-stage sampling design to enroll participants. For this purpose, it used a core national sample for African Americans and Whites, similar to the procedure applied by the National Survey of Black Americans (NSBA). This approach may be an optimal way for drawing African American samples [70,71,72]. All the NSAL participants were adults (aged 18 years and older) who were selected from households located in the coterminous 48 states. Participants were limited to the individuals who could complete a structured interview in English. Institutionalized individuals were not eligible to NSAL. As a result, individuals were excluded if they were in prisons, jails, nursing homes and long-term medical or dependent care settings [70,71,72].

### 3.4. Analytical Sample in This Study

The analytical sample in this study included a total number of 1775 employed African American adults who were either male (*n* = 676) or female (*n* = 1099). African Americans were defined as Black in the absence of any ancestral ties to the Caribbean countries.

### 3.5. Interviews and Data Collection

NSAL used structured interviews for data collection. All interviews were in English. From all the interviews, 82% were face-to-face and 14% were telephone interviews. NSAL used computer-assisted personal interviews (CAPI) for face-to-face interviews. In CAPI, the interviewer and the participant use computers to assist the process of the questions and the skip patterns. CAPI is believed to enhance the data quality at least when the survey is long and complex [73]. Interviews took on average 140 min to complete. Response rate was approximately 71% for African Americans.

### 3.6. Measures

The study measured age, gender, SES (educational attainment and household income), % Whites in workplace and perceived (daily) discrimination.

Socioeconomic Status (SES). SES was measured using two indictors: household income and educational attainment, both measured using self-reported data, via interviews. Household income was measured as (1) 0–9999 USD, (2) 10,000 USD–19,999 USD, (3) 20,000 USD–39,999 USD and (4) 40,000 USD or more. Levels of educational attainment included (1) equal or less than 11 years, (2) 12 years, (3) 13 to 15 years and (4) 16+ years. Both SES indicators were treated as continuous variables as well as dichotomous variables. As dichotomous measures, we categorized household income as (0) 0–19,999 USD versus (1) 20,000 USD or more and categorized educational attainment as (0) less than 12 years versus (1) 12 years or more. Regardless of how we treated these variables (continuous or dichotomous measures), higher educational attainment and household income indicating higher SES.

Racial Composition of Workplace. The NSAL assessed the Racial Composition of Workplace using a single item: Is your work group all Black, mostly Black, about half Black, mostly white, all white except you or what? Responses included (1) All Black, (2) Mostly Black, (3) About half Black, (4) Mostly White and (5) All White except you. This variable was treated as a continuous variable ranging from 1 to 5, with a higher score indicating higher % of Whites in the workplace.

Perceived Discrimination. Everyday discrimination in the NSAL was measured using the widely used measure of discrimination, the Everyday Discrimination Scale (EDS), developed by David Williams [74]. This scale uses 10 items to assess less overt, chronic, routine and daily discriminatory experiences occurred over the past year [74]. Example items include “In your day-to-day, life how often have any of the following things happened to you?” Sample items include: “Being followed around in stores,” “people acting as if they think you are dishonest,” “receiving poorer service than other people at restaurants,” and “being called names or insulted” [74,75,76,77]. The item responses are on a Likert scale with a potential range from 1 (never) to 6 (almost everyday). We calculated a sum score, with a range from 0 to 50, with a higher score reflecting more discrimination over the past 12 months (α = 0.86).

### 3.7. Statistical Analysis

As the NASL has used a complex sampling design, we used Stata 15.0 (Stata Corp., College Station, TX, USA) to analyze the data. We used Taylor series approximation for recalculation of the complex design-based standard errors (SE). All inferences as well as proportions and averages reported here are weighted and reflect the NSAL’s complex design. As a result, the inferences and rates are both reprehensive and generalizable to African American adults who reside in the nation. 

Survey proportions and means were used for descriptive purposes. Chi squares and independent sample *t* tests were used to compare study variables between male and female African Americans. For multivariable data analysis, structural equation modeling (SEM) was used [78]. In the first step, we fitted a model to the pooled sample. Then we ran multi-group model where groups were based on gender. We ran models with and without constrained paths across the groups. As constraints did not change the model fit, we reported the models without constrains as final models. We compared the path coefficients between the groups for statistically significant difference. As the data were weighted, most conventional fit indices [79,80,81] were not available. Full information maximum likelihood (FIML) was used to handle missing data. Adjusted path coefficients (b) and their associated 95% confidence intervals (CIs) and *p*-values were reported. A *p*-value of smaller than 0.05 was considered as statistically significant [82].

## 4. Results

### 4.1. Descriptive Statistics

This study included a total number of 1775 African American adults who were in the labor market. These participants were either male (*n* = 676) or female (*n* = 1099). Table 1 describes age, SES (household income and educational attainment), racial composition of the workplace and perceived discrimination overall and by gender. Educational attainment was higher in females but income was more in African American men. Perceived discrimination was also higher in African American males than females. Age was not different between male and females.

### 4.2. SEM in the Pooled Sample, No Mediator

**Figure 1:**
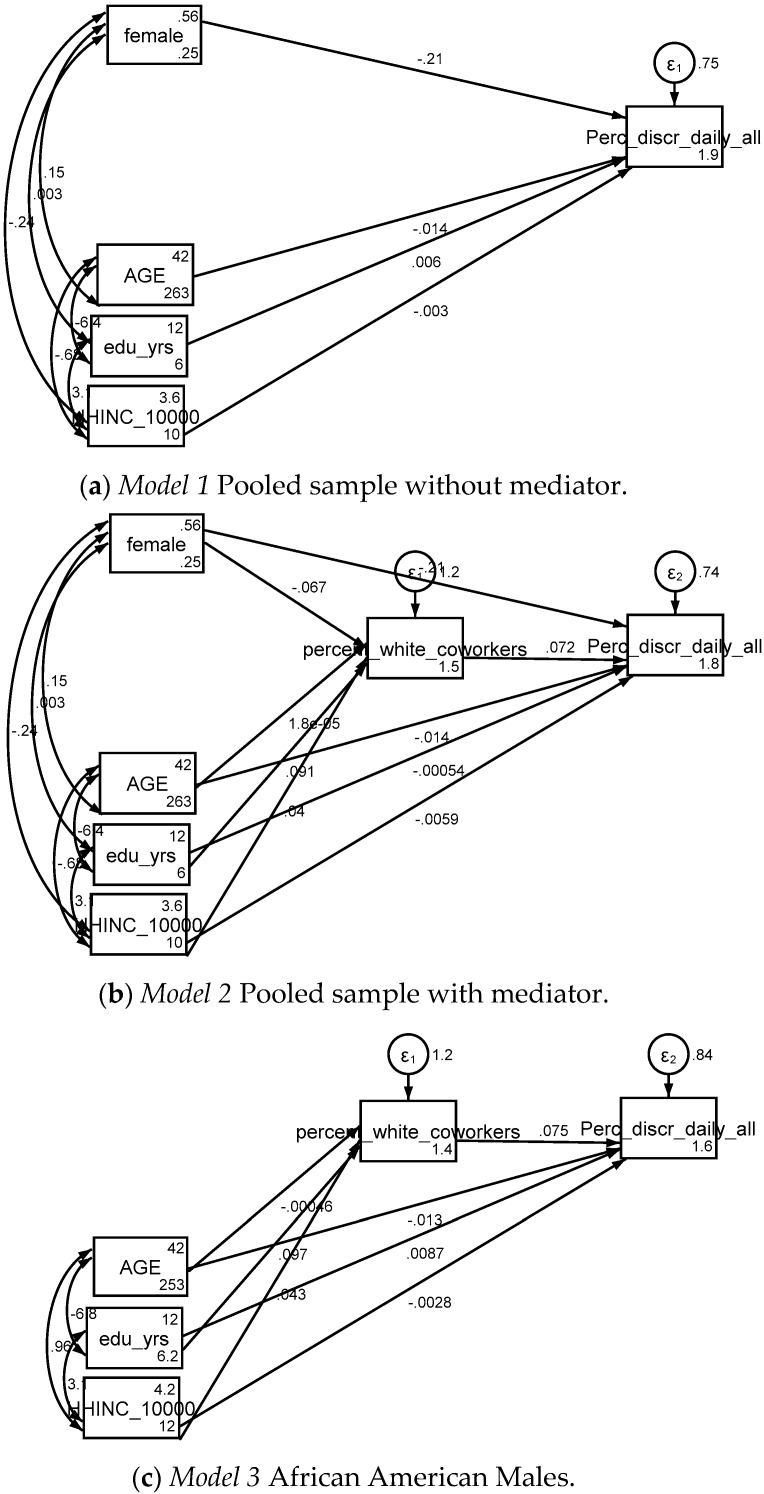

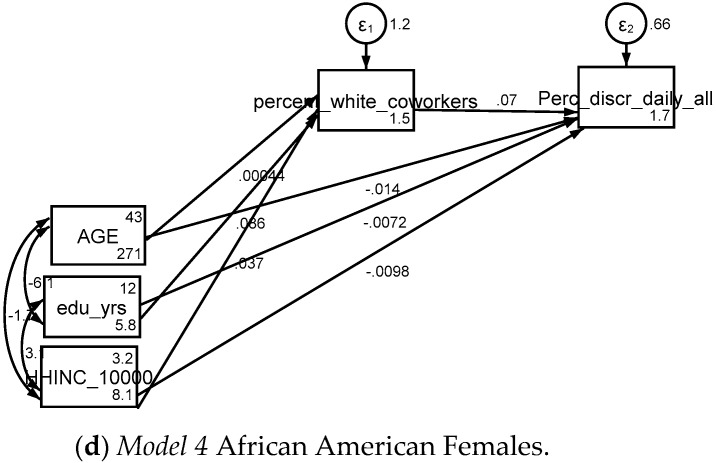
Summary of path model on the associations between socioeconomic status (household income and educational attainment), racial composition of workplace and perceived discrimination in employed African American men and women. (**a**) *Model 1* Pooled sample without mediator; (**b**) *Model 2* Pooled sample with mediator; (**c**) *Model 3* African American Males; (**d**) *Model 4* African American Females. Notes: Outcome: Discrimination (Everyday), Independent variables treated as continuous measures, Household income measured as (1) 0–9999 USD, (2) 10,000 USD–19,999 USD, (3) 20,000 USD–39,999 USD and (4) 40,000 USD or more. Educational attainment measured as (1) equal or less than 11 years, (2) 12 years, (3) 13 to 15 years and (4) 16+ years. Confidence Interval (CI); Standard Error (SE); * *p* < 0.05, *** *p* < 0.001.

Table 2 summarizes the results of an SEM with perceived discrimination as the outcome, SES indicators as continuous measures as the independent variables and age and gender as the covariates. *Model 1* was estimated in the pooled sample in the absence of racial composition of workplace as the mediator. Based on *Model 1*, in the pooled sample, higher level of educational attainment and household income were associated with more perceived discrimination (Figure 1a, Table 2). 

### 4.3. SEM in the Pooled Sample, With Mediator

Table 3 provides a summary of the results of the second SEM. *Model 2* also included racial composition of workplace as the mediator of the association between SES and perceived discrimination. Based on *Model* 2, educational attainment and income were associated with a higher % of Whites in the workgroup, which was in turn associated with higher perceived discrimination (Figure 1b, Table 3).

### 4.4. SEM by Gender; Stratified Models

Multi-group SEM did not show any differences by gender. So, high SES is associated with working in a predominantly White workplace, which in turn is associated with more perceived discrimination, regardless of gender (Figure 1c,d).

## 5. Discussion

The current study tested whether gender alters the pattern of associations between SES indicators (i.e., household income and educational attainment), racial composition of workplace and perceived discrimination. Using a nationally representative sample of African American adults, two major results were found. First, both household income and educational attainment were positively associated with working in a majority White area, which was in turn associated with more perceived discrimination. Second, these associations did not vary between African American men and African American women. That is, gender does not seem to moderate the effect of SES racial composition of workplace and its effect on perceived discrimination.

These findings suggest that working in predominantly White workplaces may explain why high SES African Americans report more discrimination and poor mental health. These results are related to a growing scientific literature on the intersection of race/ethnicity, gender and class perceived discrimination and mental health. Worse mental health of high SES African Americans, particularly African American men, is reported and perceived discrimination may explain this pattern [12,15,16,17,19,20]. However, the current study did not reveal gender differences in the role of racial composition of workplace as a potential mechanism for why male gender is a vulnerability factor for discrimination. This study, however, provides a partial explanation for why SES operates as a vulnerability factor for African Americans. 

To give an example, in the NSAL-Adolescents study, high household income was associated with higher risk of lifetime, 12-month and 30-day MDD among male African American youth [16]. In a longitudinal study with 18 years of follow up, African American youth from high income families and those who were living in predominantly White areas reported higher levels of discrimination between ages 9 to 37 years, compared to their low SES counterparts and those who lived in majority Black areas [23]. In another analysis of the same data (over the same 18 years of follow up), African American youth from high SES families and those who were living in predominantly White areas were more depressed, an association which was fully explained by perceived discrimination [24]. 

The results propose that interracial anxiety and workplace discrimination may have some role in explaining diminished gain of SES in African Americans. 

In addition to the distribution of discrimination, gender and SES also alter the consequences of discrimination. Male African Americans, particularly those from high SES, seem to be at a disadvantage compared to their female and low SES counterparts for the sensitivity/vulnerability to discrimination [23]. In an 18-year study of African American youth, an incremental increase in perceived discrimination over one year was associated with an increase in symptoms of anxiety and depression a decade later for male but not female African American youth/young adults [42]. This pattern of extra-vulnerability of males to discrimination is also found in other ethnic groups [43] and is shown for other outcomes such as substance use [83]. In another study of African American youth, high subjective SES was associated with an increase in the effect of perceived discrimination on risk of MDD [25]. 

As a result, the racial composition of a workplace may have a similar role for high perceived discrimination of high SES male and female African Americans. These findings are relevant to the high costs of upward social mobility as reported by other researchers [19,20,22]. Fuller-Rowell et al., [84] and others on the intersection of race, upward social mobility and health. Fuller-Rowell et al., found a weaker protective effect of educational attainment on the health of African American than White youth [84]. High discrimination of African American males also explains African Americans’ diminished health gain from SES [85]. In the ACL data, high education credentials at baseline were predictive of an increase in depressive symptoms over a 25-year follow up for African American men. At the same time, for African American males, each additional year of schooling was protective against depression, suggesting that the observed diminished returns of SES are due to labor market practices rather than individual behaviors and choices [15]. John Henryism, which is commonly used by African American men for upward social mobility, may be a reason high SES adds to the psychological costs for this group [86,87,88].

## 6. Directions for Future Research

More research is needed before we know whether perceived discrimination is why SES resources generate less health gains or sometimes increased mental health risk for African American men. Future research may include racial attitudes, gender norms, attribution style, race socialization, social support, ethnic identity and coping styles that may have a role in the differences in exposure and vulnerability of African American males to discrimination at each SES level. Research should also test racial composition at work and differential discrimination from various sources. Future research could test the role of work related contextual factors such as percent of various races in explaining some of the discrimination reported by high SES African America males and females in this study. Finally, biological studies should test whether chronic discrimination and stress operate similarly or differently in impacted brain regions and networks that are impacted by depression.

Fundamental Cause Theory (FCT) and Social Determinants of Health (SDH) suggest that health gain follows SES resources and upward social mobility is linked to health. While this general argument is still true [50,51,52], SES may operate as a vulnerability factor for minority populations [17,26] and whether SES operates as a protective or vulnerability status may be relevant to some but not all groups based on the intersection of multiple social identities (e.g., race, ethnicity, gender, class and place) [12,26]. Psychosocial gain and harm of upward and downward social mobility may depend on how easy or hard it to be an upwardly mobile under a certain skin color and gender and place. We know that upward social mobility has different effects on the health of population subgroups [84]. One study with old data suggested that downwardly mobile Whites and upwardly mobile Blacks are at higher risk of mental distress than other groups [89].

## 7. Limitations

There are a few study limitations. First, because of the cross-sectional design of the study, we cannot interpret the observed associations as causations. Second, the study did not have a balanced sample size for male and female groups. This may have resulted in differential statistical power. Third, the current study only controlled for a few confounders such as age. Important information about participants’ experiences at work—the number of years employed, their job status (i.e., managerial or not), occupational prestige, hours employed and employment history—were missing. Other psychological factors such as race socialization, racial identity, personality, coping and vigilance were also not measured. Finally, the racial composition of neighborhoods and area level SES could provide additional relevant information. This is particularly important because the data were fairly old (i.e., collected in 2003) and there may have been changes in expectations and workplace diversity since then. Thus, there is a need to replicate these findings using more recent data. Despite these limitations, the current study still contributes to the literature as very few studies have documented a positive link between gender, SES and perceived discrimination. These results are suggestive that the racial composition of a workplace may be a mechanism linking SES to perceived discrimination, however, similarly for African American men and women.

## 8. Conclusions

To summarize, these findings suggest that SES (education and household income) change the racial composition of the workplace and high SES is associated with an increased exposure of African American workers to Whites in the workplace, which is in turn associated with more perceived discrimination. The workplace may explain why SES resources are associated with lower health gains and more perceived discrimination for African American men and women. These findings provide a potential explanation for why high SES African American men and women gain less health and why high SES and male African Americans are at high risk for poor mental health from their SES resources [21,54] and are at high risk of depression [12,15,16,17,19,20].

## Figures and Tables

**Table 1:** Descriptive statistics in the pooled sample of employed African Americans.

	African Americans (*n* = 1775)		African American Men (*n* = 676)		African American Women (*n* = 1099)	
	Mean (SE)	95% CI	Mean (SE)	95% CI	Mean (SE)	95% CI
Age (Years)	37.77 (0.44)	36.88–38.66	37.88 (0.62)	36.63–39.13	37.67 (0.55)	36.54–38.79
Education (Years)	12.87 (0.08)	12.69–13.04	12.73 (0.12)	12.49–12.98	12.99 (0.10)	12.79–13.19
Educational attainment (1–4) *	2.42 (0.04)	2.35–2.49	2.36 (0.05)	2.26–2.46	2.47 (0.05)	2.37–2.57
Household Income (1–5) *	4.22 (0.15)	3.91–4.53	4.66 (0.22)	4.21–5.11	3.80 (0.15)	3.50–4.11
% Whites in Workplace	2.22 (0.05)	2.11–2.32	2.18 (0.07)	2.04–2.31	2.25 (0.05)	2.14–2.36
Perceived Discrimination (Everyday) *	1.31 (0.04)	1.24–1.39	1.41 (0.05)	1.30–1.52	1.23 (0.04)	1.15–1.30

Notes: Household income measured as (1) 0–9999 USD, (2) 10,000 USD–19,999 USD, (3) 20,000 USD–39,999 USD and (4) 40,000 USD or more. Educational attainment measured as (1) equal or less than 11 years, (2) 12 years, (3) 13 to 15 years and (4) 16+ years. Confidence Interval (CI); Standard Error (SE); * *p* < 0.05 for comparison of men and women.

**Table 2:** Summary of path coefficients for the effects of household income and educational attainment on perceived discrimination via racial composition of workplace in the pooled sample.

			b (SE)	95% CI	*p*
			*Model 2* (All + Mediator)		
Gender (Female )	→	% White coworkers	−0.07 (0.06)	−0.19–0.06	0.289
Age	→	% White coworkers	0.00 (0.00)	−0.01–0.01	0.994
Education (Years)	→	% White coworkers	0.09 (0.02)	0.06–0.12	0.000
Income (1000 USD)	→	% White coworkers	0.04 (0.01)	0.02–0.06	0.001
% White coworkers	→	PD	0.07 (0.02)	0.03–0.12	0.002
Gender (female)	→	PD	−0.21 (0.04)	−0.28–0.14	0.000
Age	→	PD	−0.01 (0.00)	−0.02–0.01	0.000
Education (Years)	→	PD	0.00 (0.01)	−0.02–0.01	0.943
Income (1000 USD)	→	PD	−0.01 (0.01)	−0.02–0.01	0.320

Notes: Outcome: Discrimination (Everyday), Independent variables treated as continuous measures, Household income measured as (1) 0–9999 USD, (2) 10,000 USD–19,999 USD, (3) 20,000 USD–39,999 USD and (4) 40,000 USD or more. Educational attainment measured as (1) equal or less than 11 years, (2) 12 years, (3) 13 to 15 years and (4) 16+ years. Confidence Interval (CI); Standard Error (SE); Perceived Discrimination (PD); b, Adjusted path coefficient.

**Table 3:** Summary of path coefficients for the effects of household income and educational attainment on perceived discrimination via racial composition of workplace in the pooled sample.

			b (SE)	95% CI	b (SE)	95% CI	b (SE)	95% CI
			*Model 1* (All–Mediator)		*Model 2* (Males–Mediator)		*Model 3* (Males–Mediator)	
Gender (female )	→	PD	−0.20 (0.05) ***	−0.30–0.11				
Age	→	PD	−0.01 (0.00) ***	−0.02–0.01	−0.01 (0.00) ***	−0.02–0.01	−0.01 (0.00) ***	−0.02–0.01
Education (Years)	→	PD	0.03 (0.01) **	0.01–0.06	0.04 (0.02) *	0.00–0.07	0.03 (0.01) *	0.00–0.06
Income (1000 USD)	→	PD	−0.01 (0.01)	−0.02–0.01		−0.02–0.01	−0.01 (0.01)	−0.03–0.01

Notes: Outcome: Discrimination (Everyday), Independent variables treated as continuous measures, Household income measured as (1) 0–9999 USD, (2) 10,000 USD–19,999 USD, (3) 20,000 USD–39,999 USD and (4) 40,000 USD or more. Educational attainment measured as (1) equal or less than 11 years, (2) 12 years, (3) 13 to 15 years and (4) 16+ years. Confidence Interval (CI); Standard Error (SE); Perceived Discrimination (PD). b, Adjusted path coefficient.
